# Supplementary material for: Content-rich biological network constructed by mining PubMed abstracts
Source: BMC Bioinformatics. 2004 Oct 8;5:147. doi: 10.1186/1471-2105-5-147 (PMC528731; doi:10.1186/1471-2105-5-147)
Supplement: Additional File 2 — The original results of the above study (non-essential files are deleted to keep the file size under the limit set by BMC bioinformatics). [file 1471-2105-5-147-S2.bz2 › chilibotAdditionalFile2/dip05/193ID7644522E674/html/ECR_RXRA.html]

 


 **ECR** and **RXRA** 
  
Found 1 abstracts in PubMed, retrieved 1.  
 

 What does Google say? 
 PDF only 
| .edu only 

---

**Interactive relationship** (e.g. stimulation, inhibition, etc)

**Neutral relationship**- Human embryonic kidney HEK 293 cells expressing the regulatory vector pVgRXR  **EcR**  293 cells were transfected with rat NR1a and NR2B  [ **RXRA** ]  cDNAs using the inducible vector pIND Invitrogen.  Ref: 12605879 Neurochem Int, 2003

**Non-interactive relationship** (e.g. studied together, co-existance, homology, etc.)

- The pharmacological profile of the expressed NMDA receptors was characterised by comparing the inhibitory activity of several NR2B  [ **RXRA** ]  subunit selective NMDA antagonists in  **EcR**  rNR1a2B cells with that observed in primary cultures of rat cortical neurones.  Ref: 12605879 Neurochem Int, 2003
- Inducible expression of the NR2B  [ **RXRA** ]  subunit in cell clone designated  **EcR**  rNR1a2B was investigated using quantitative RT PCR and flow cytometry based immunocytochemical methods.  Ref: 12605879 Neurochem Int, 2003
- The mRNA level of the NR2B  [ **RXRA** ]  subunits in  **EcR**  rNRa2B cells was dependent on the concentration of the ecdysone analogue inducing agent, muristerone A MuA.  Ref: 12605879 Neurochem Int, 2003
- Whereas the efficacies of the NR2B  [ **RXRA** ]  subunit selective NMDA antagonists were similar in  **EcR**  rNR1a2B cells and in neurones, their maximal inhibitory effects were significantly higher in cells expressing NR1a NR2B  [ **RXRA** ]  recombinant receptors.  Ref: 12605879 Neurochem Int, 2003
